# Identifying the subgroups of depression trajectories among the middle-aged and older Chinese individuals with chronic diseases: an 8-year follow-up study based on CHARLS

**DOI:** 10.3389/fpubh.2024.1428384

**Published:** 2024-09-11

**Authors:** Jiaxing Pei, Mei Hu, Qiang Lu, Pengfei Zhou, Yijing Shang, Huiwang Zhang, Xiaoguang Yang, Yunming Li

**Affiliations:** ^1^Department of Information, Medical Support Center, The General Hospital of Western Theater Command, PLA, Chengdu, China; ^2^School of Public Health, Southwest Medical University, Luzhou, China; ^3^Department of Outpatient, The General Hospital of Western Theater Command, PLA, Chengdu, China; ^4^Department of Health Economics, The General Hospital of Western Theater Command, Chengdu, China

**Keywords:** depression, aging, chronic disease, latent class mixture modeling, trajectories

## Abstract

**Background:**

Prior studies have demonstrated a prevalent occurrence of depression among the middle-aged and older Chinese individuals with chronic diseases. Nevertheless, there is limited research on the specific subgroups of depression trajectories within this population and the factors influencing these subgroups.

**Objective:**

To explore the changing trajectory and influencing factors of depression in the middle-aged and older individuals with chronic disease in China, and provide the data reference for the health management of the older adult population in China.

**Methods:**

A longitudinal cohort study was conducted using the data from the China Health and Retirement Longitudinal Study (CHARLS) in 2011, 2013, 2015, 2018, and 2020. A total of 2,178 participants with complete data were included. The level of depression was evaluated using the Center for Epidemiologic Studies Depression Scale (CESD-10). The Latent Class Mixed Models (LCMM) were employed to estimate trajectories of depressive symptoms. The Kruskal-Wallis *H* test and the Pearson *χ*^2^ test were used to determine the significant factors affecting trajectory grouping. Subsequently, the multinomial logistic regression model was utilized to perform a multifactorial analysis of the variables impacting the trajectory subgroup of change in depressive symptoms.

**Results:**

The LCMM-analysis revealed three distinct subgroups of depression trajectories: the “Low stable group” comprising 36.7% of the sample, the “Medium growth group” comprising 34.4% of the sample, and the “High growth group” comprising 28.9% of the sample. Among the baseline characteristics of different depression trajectory subgroups, there were significant differences in gender, residence, education, marital status, social activity participation, number of chronic diseases, smoking status, BMI, midday napping (minutes) and nighttime sleep duration (hours). Through multiple logistic regression analysis, our findings demonstrate that among the middle-aged and older Chinese individuals with chronic diseases, the following individuals should be the key groups for the prevention and treatment of depressive symptoms: Those who are young, female, residing in rural areas, having primary school education and below, being single, not participating in social activities, suffering from multiple chronic diseases, and having shorter naps and sleeping at night.

**Conclusion:**

There is heterogeneity in the subgroups of depression trajectories among the Chinese middle-aged and older individuals with chronic diseases. The focus should be on the distinct characteristics of various trajectories of depression within the realm of health management.

## 1 Introduction

The Chinese government is currently grappling with the issue of an aging population that is estimated to surge in numbers dramatically by 2050, with individuals aged 65 and above reaching 400 million, accounting for 26.9% of the total population (1). Since 2015, China’s life expectancy has shown an upward trend. According to China’s General Program for Sustainable Development, the life expectancy will reach 85 years by 2050 (2). Moreover, the demographic shift to aging could lead to an increase in the number of people living with chronic diseases, as aging is the largest risk factor for the incidence of most chronic diseases (3). A survey by Su et al. (4) aimed at revealing the epidemiological characteristics of chronic diseases among the older adults in China found that about 180 million older adults in China suffered from chronic diseases in 2015. A study by Islam et al. (5) found that a diagnosis of any chronic disease leads to an increased burden on mental health, including depression, psychological distress, and post-traumatic stress disorder. Depression is a mental health disorder, characterized by a range of symptoms including low mood, slowed thinking, reticence, and in severe cases, suicidal tendencies (6). A meta-analysis by Lim et al. (7) covering 30 countries looked at the prevalence of depression in community populations between 1994 and 2014. The study found that South America had the highest overall prevalence at 20.6 percent, followed by Asia at 16.7 percent, North America at 13.4 percent, Europe at 11.9 percent and Africa at 11.5 percent. Australia had the lowest prevalence of depression at 7.3 percent. Another study by Ren et al. (8) indicated that China’s population constitutes 18.4% of the global population, while the number of individuals suffering from depression in China makes up 21.3% of the world’s total, which highlights depression as a significant public health challenge in China.

A substantial body of evidence from multiple studies has demonstrated the correlation between chronic diseases and depression (9–11). A survey by Moussavi et al. (12) covering 60 countries revealed that the participants with one or more chronic diseases exhibited a prevalence of depression ranging from 9.3 to 23.0%, compared to an average of 3.2% among those without chronic diseases. Therefore, the result indicates that participants with chronic diseases are more likely to experience depression. While most studies on the relationship between depression and chronic diseases are based on cross-sectional surveys, a recent study by Wang et al. (13) used longitudinal data to investigate the correlation among the middle-aged and older Chinese. Our previous longitudinal study, based on the CHARLS database, demonstrated that the risk of depression in the middle-aged and older adult population increases with the number of chronic diseases they have (14). Yet, many studies, including ours, have overlooked the trajectories of different depressive symptoms in this demography, and even fewer have explored the trajectory changes of depression among those with chronic disease. Depression trajectory refers to the use of trajectory analysis methods to describe and classify different patterns of depression symptom development over time based on longitudinal data of individual depression symptoms (15). By analyzing the different subgroups of depression trajectories, researchers can better understand the long-term development of depression, identify high-risk groups, and provide scientific basis for personalized treatment and prevention programs. Hence, exploring the different subgroups of depression trajectories among the middle-aged and older Chinese individuals with chronic diseases can be instrumental in preventing or delaying the onset of depression.

The Latent Class Mixed Model (LCMM) has been used in previous studies to identify subgroups of depression trajectories (16, 17). This model which is an extension of the standard linear mixed model, does not predefine subgroups’ depression trajectories based on observers’ characteristics. Instead, it captures heterogeneity in individual trajectories and distinguishes subgroups with similar trajectory features. The model considers inter-category heterogeneity and intra-category individual effects, leading to a better fitted model (18).

Based on this, our research participants consist of the middle-aged and older Chinese individuals aged 45 years or older with chronic diseases (19), and the intent is to use the LCMM to reveal the diversity of depressive trajectories and analyze factors influencing these trajectories. This may help in developing personalized intervention and treatment strategies according to the depression trajectories.

## 2 Materials and methods

### 2.1 Population

The data for this study was obtained from the China Health and Retirement Longitudinal Study (CHARLS). This survey was funded by Peking University. Its objective was to collect comprehensive, high-quality public microdata from a representative sample of individuals and families in China. The data is employed to analyze the ageing situation of Chinese population, to promote interdisciplinary research on ageing issues, and to provide a more scientific basis for the formulation and improvement of relevant policies in China. In order to ensure the representativeness of the data, the CHARLS employed stratified sampling and probability proportional sampling in 150 counties and 450 communities (villages) across 28 provinces (autonomous regions and municipalities) in 2011, 2013, 2015, 2018, and 2020, respectively. Consequently, the CHARLS data is considered to be broadly representative and reflects the overall situation of middle-aged and older adults in urban and rural China (20).

**Figure 1:**
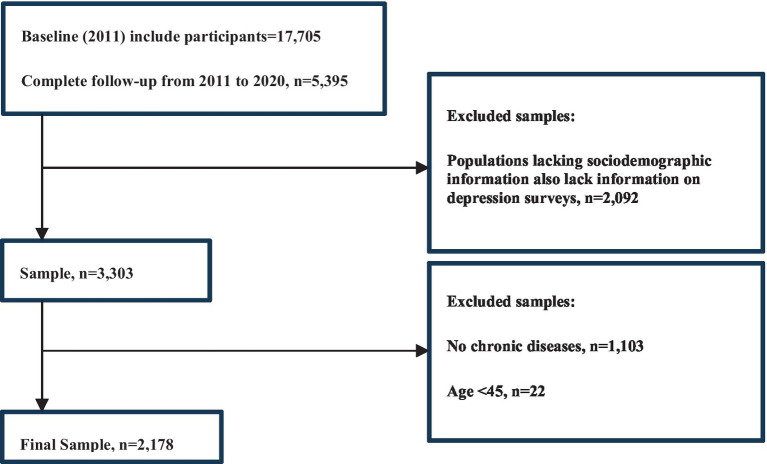
Process of sample population screening.

Our research included participants suffering from the following 14 categories of chronic diseases: hypertension; dyslipidemia (elevation of low density lipoprotein, triglycerides, and total cholesterol, or a low high density lipoprotein level); diabetes or high blood sugar; cancer or malignant tumor (excluding minor skin cancers); chronic lung diseases, such as chronic bronchitis, emphysema (excluding tumors, or cancer); liver disease (excluding fatty liver, tumors, and cancer); heart attack, coronary heart disease, angina, congestive heart failure, or other heart problems; stroke; kidney diseases (excluding tumors or cancer); stomach or other digestive disease (excluding for tumor or cancer); emotional, nervous, or psychiatric problems; memory-related disorders (such as dementia, cerebral atrophy, Parkinson’s disease); arthritis or rheumatism; asthma. We used data from five waves of the survey conducted in 2011, 2013, 2015, 2018 and 2020. The baseline data in 2011 included 17,705 people. A cohort of 5,395 people were followed up from 2011 to 2020. Of these individuals, 1,103 were excluded because they did not have chronic diseases and 2,092 were excluded because essential baseline information (such as age, gender, education, marital status, etc.) was missing. In addition, 22 participants were excluded because they were under 45 years of age. In total, 2,178 participants were selected for analysis (Figure 1).

### 2.2 Measurement of depression

The depression of the study participants was evaluated using the 10-item Center for Epidemiologic Studies Depression Scale (CESD-10). The CESD-10 scale has good internal consistency (Cronbach *α* = 0.7 to 0.9), sensitivity (71.4 to 84.6%) and specificity (72.6 to 95.0%) for screening depressive symptoms (21). It has been fully validated in the Chinese older adult population to demonstrate its reliability and validity (22, 23). In detail, the scale comprised 10 questions which were designed to assess the psychological state of the respondent over the previous week. The depressive symptom score of participants was derived from the total score of the above 10 items and ranged from 0 to 30, with higher score indicating higher levels of depressive symptoms, and the participants were classified as depressed individuals if their total score was 10 or greater (21).

### 2.3 Measurement of the number of chronic diseases

The participants were asked about the diagnoses they had received from their physicians. For those with a chronic illness, a scoring system was utilized: a score of 1 was given if participants confirmed having the condition, while a score of 0 was recorded if they did not report the condition. By summing these scores, the total number of chronic conditions per participant was calculated.

### 2.4 Other covariates

The following characteristics of the study participants at baseline were compare (detailed in Supplementary Appendix 1): age, gender (female, male), residence (urban, rural), education (primary school and below, junior high school, high school and above), marital status (married, single), health insurance status (no, yes), physical activity (no, yes), social activity participation (no, yes), and smoking status (never, former, current), drinking status (no, yes), body mass index (BMI), midday napping (minutes), nighttime sleep duration (hours) (24–26). The types of health insurance in the original survey data were categorized as urban and rural health insurance, long-term care insurance, urban workers’ health insurance, private health insurance, government health insurance, etc. For the purposes of this study, the participants were considered to be insured if they held any of these types of health insurance. The social activity participation was categorized as hanging out, socializing with friends, attending community events, and participating in activities organized by clubs. The participants were considered to have exhibited social behaviors if they had engaged in any of these types of social activities in the past month; physical activities included cycling, tai chi, walking, etc. The participants were considered to be physically active if they performed the above activities on a weekly basis. BMI was calculated by dividing weight (in kilograms) by height squared (in meters squared), with respondents’ weight and height obtained from on-site measurements (27). The participants were requested to provide information regarding the duration of their naps (in minutes) over the past month and their average nighttime sleep duration (in hours) over the past month.

### 2.5 Statistical analysis

#### 2.5.1 Trajectory identification of depression subgroups

We used R 4.2.1 and SPSS 16.0 software for statistical analyses. Subgroups of depression trajectories over time were identified using the “LCMM” package in R. The CESD-10 score at the 2011, 2013, 2015, 2018, and 2020 time points were used for trajectory modeling, with 1 to 4 classes being fitted in the process of model construction. Each model with two or more classes used random starting values from the model with the 1-class. We primarily identify the optimal trajectory model using the Bayesian information criterion (BIC), reference Akaike Information criterion (AIC), the sample size-adjusted Bayesian information criterion (SABIC), and Entropy. We then determine the posterior probability based on class (>0.70) and class size (≥2% of the population) (28).

#### 2.5.2 Analyze of factors influencing subgroups of depressive symptoms trajectories

After modeling the trajectories, we have expressed them as means and standard errors to better illustrate whether the sample is representative of the population; the prevalence of depression is expressed as *p* (rate) and 95% *CI* (confidence interval). Subsequently, non-parametric tests were employed for analysis. The Kruskal-Wallis *H* test was utilized for comparing multiple subgroups, while percentage representation was used for enumeration data. The subgroup comparisons were conducted using the Pearson *χ*^2^ test. After identifying significant factors affecting trajectory grouping, the multinomial logistic regression model was adopted to perform a multifactorial analysis of the factors influencing the trajectory of change in depressive symptoms. To assess the robustness of the outcomes, we conducted a sensitivity analysis. The principal analysis was replicated for middle-aged and older adults with five waves of depression, including the middle-aged and older Chinese individuals without chronic diseases.

A significance level of *p* < 0.05 was applied to determine statistical significance in this academic context.

## 3 Results

### 3.1 Descriptive statistics

**Figure 2:**
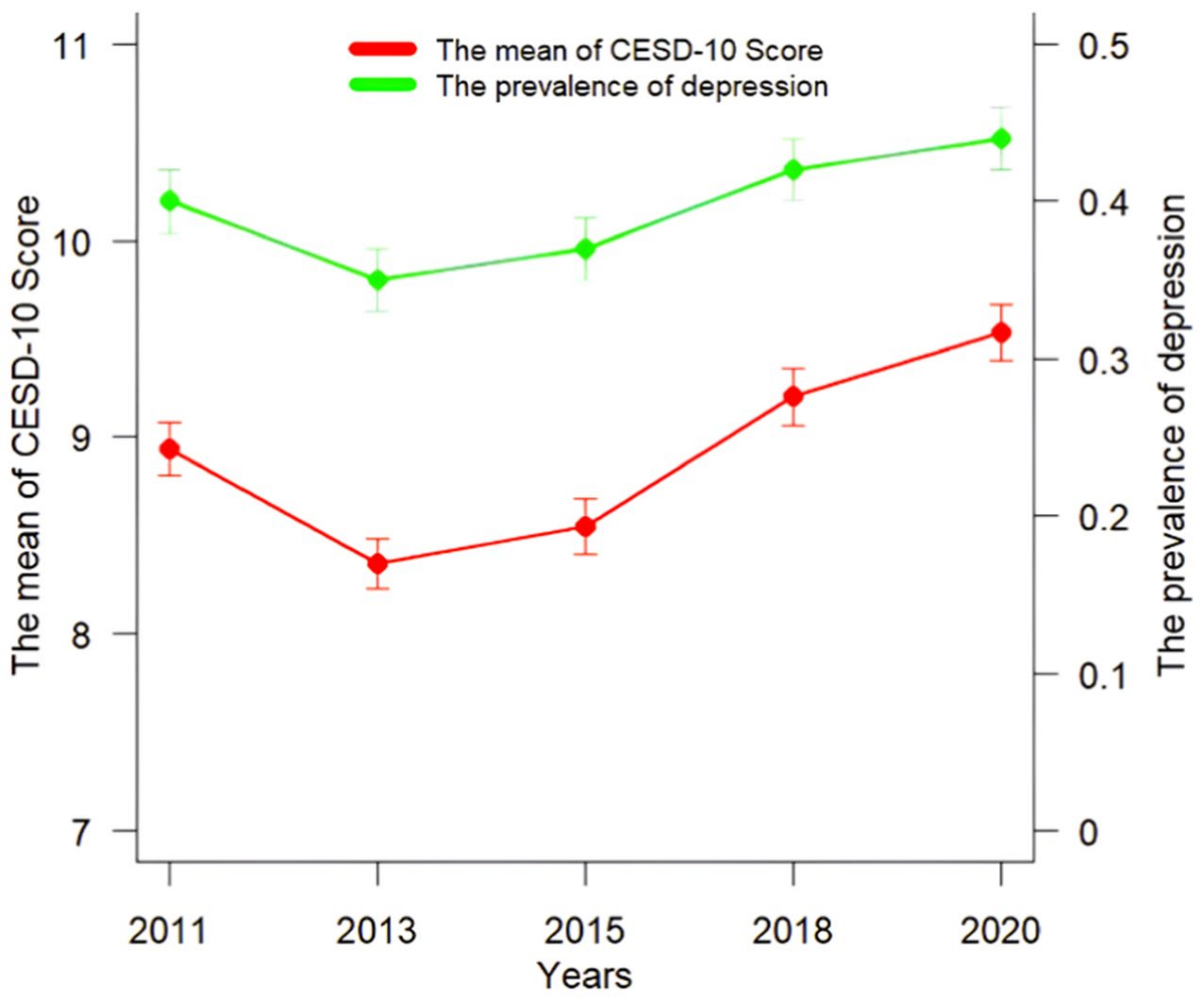
The mean of CES-D score (mean ± standard error) across 5 data collections during 2011–2020; 12-month prevalence (*p* 95% *CI*).

In a long-term observation of CESD-10 score in the middle-aged and older Chinese individuals with chronic diseases, the data shows a general upward trend. Specifically, from an initial mean score of 8.94 ± 0.14 in 2011 to 8.36 ± 0.13 in 2013, the score then starts to increase year on year, rising to 8.55 ± 0.14 in 2015 and then further increasing to 9.21 ± 0.15 in 2018, reaching 9.53 ± 0.14 in 2020. This suggests that the CESD-10 score of Chinese middle-aged and older individuals with chronic diseases shows a slight initial decline, followed by an increase during the observation period. About the observation of the prevalence of depression in the Chinese middle-aged and older individuals with chronic diseases, the data also shows a general upward trend. In detail, the prevalence of depression decreases from 40.73% (38.65, 42.82%) in 2011 to 35.31% (33.30, 37.36%) in 2013, since then the prevalence shows an upward trend to 36.87% (34.84, 38.93%) in 2015, and further increases to 42.06% (40.00, 44.16%) in 2018 until 2020 when it reaches 43.66% (41.57, 45.78%) (Figure 2).

In summary, the mean of CSD-10 score and the prevalence of depression in the middle-aged and older Chinese patients with chronic diseases show an overall upward trend from 2011 to 2020. It is necessary to analyze the trajectory of changes in depressive symptoms and the factors influencing them in this population.

### 3.2 Subgroups of depression trajectories outcomes

**Figure 3:**
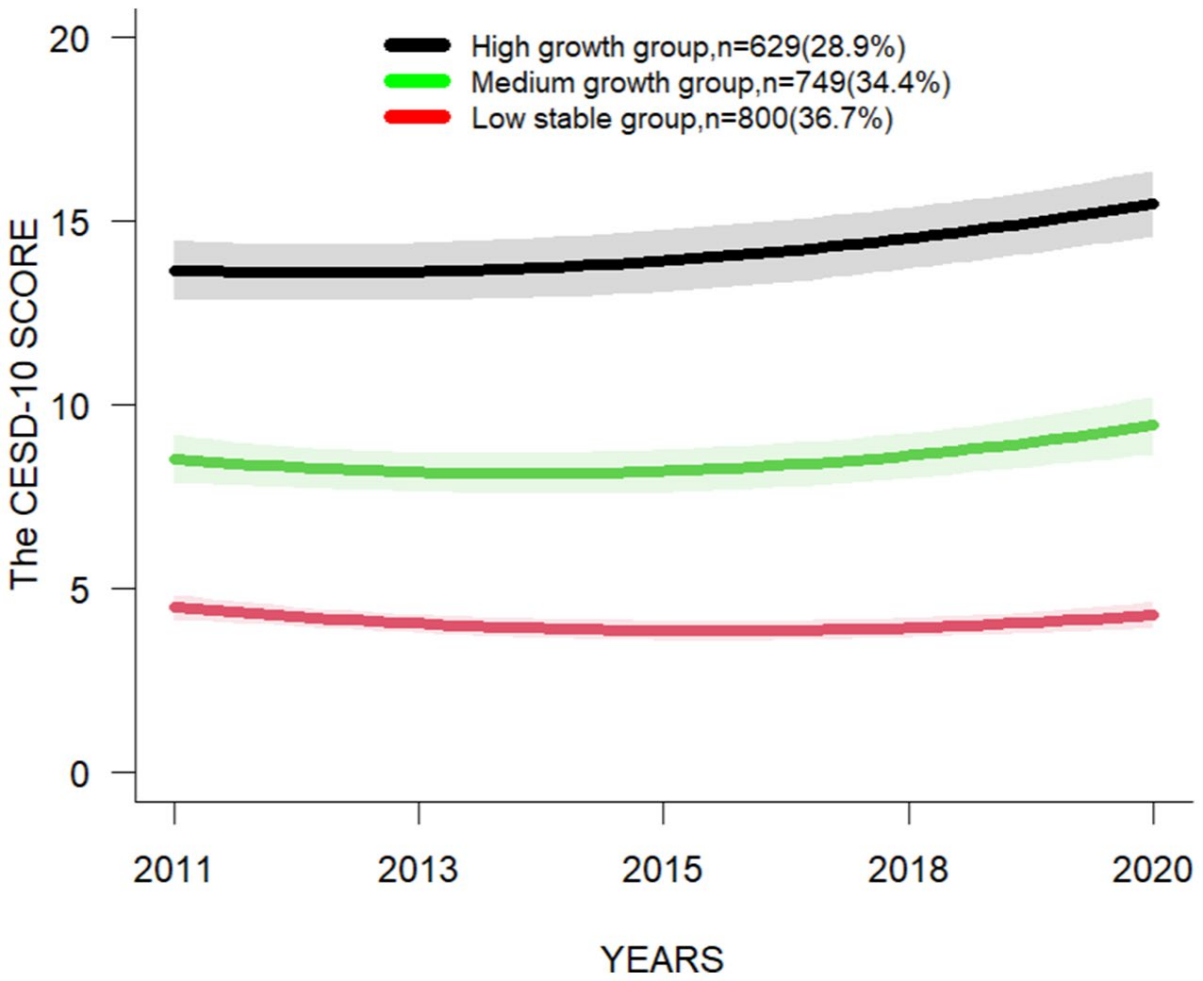
Longitudinal LCMM based depression trajectories using CESD-10 score.

Four LCMM models were selected in the following order: single-trajectory, dual-trajectory, triple-trajectory, and quadruple-trajectory models (Supplementary Table S1), and the model with the lowest BIC value is the preferred mode (29). Besides, considering the indicators of the model and the simplicity, accuracy and practical significance of the model, it is the most reasonable to choose the triple-trajectory classification model (Figure 3). The chosen model integrates the trajectory of three interregional changes in CESD-10 scores within the target population.: the mean of CESD-10 score of the “Low stable group “is below 4.5 at all-time points, indicating a stable trend; the mean of CESD-10 score of the “Medium growth group “is close to 10 at all-time points, but does not exceed 10, and shows an increasing trend; and the mean of CESD-10 score of the “High growth group “is above 10 at all-time points, indicating a growth trend (Table 1).

**Table 1:** The mean of CESD-10 score and the prevalence of different depression trajectory subgroups at various time points.

Trajectory subgroups		2011	2013	2015	2018	2020
Low stable group *n* = 800	The mean of CSED-10 score (Mean ± SE)	4.37 ± 0.12	4.02 ± 0.10	3.52 ± 0.10	3.85 ± 0.11	4.12 ± 0.11
	The prevalence of depression (*p*, 95%CI)	7.50 (5.77,9.55)	3.88 (2.65,5.46)	4.38 (3.07,6.03)	6.00 (4.46,7.88)	5.63 (4.13,7.45)
Medium growth group *n* = 749	The mean of CSED-10 score (Mean ± SE)	8.90 ± 0.16	8.15 ± 0.15	8.23 ± 0.15	9.17 ± 0.17	9.48 ± 0.15
	The prevalence of depression (*p*, 95%CI)	44.73 (41.13,48.37)	34.58 (31.17,38.11)	34.85 (31.43,38.38)	45.13 (41.52,48.77)	48.46 (44.83,52.11)
High growth group *n* = 629	The mean of CSED-10 score (Mean ± SE)	14.80 ± 0.26	14.12 ± 0.23	15.32 ± 0.25	16.07 ± 0.25	16.48 ± 0.24
	The prevalence of depression (*p*, 95%CI)	78.22 (74.79,81.39)	76.15 (72.62,79.43)	80.60 (77.29,83.62)	84.26 (81.18,87.02)	86.33 (83.39,88.92)

### 3.3 Basic characteristics of subgroups of the depression trajectories

The results of the baseline characteristics and univariate analyses of the middle-aged and older chronic disease individuals grouped according to different trajectories of depressive symptom development are shown. The results showed that the subgroups were statistically significant (*p* < 0.05) in terms of gender, residence, education, marital status, social activity participation, number of chronic diseases, smoking status, BMI, midday napping, and nighttime sleep duration (Table 2).

**Table 2:** The statistical differences in influence factors among the different subgroups of depression trajectories.

Variables		Baseline characteristics *n* = 2,178	Trajectory subgroups			H/*χ*^2^	*p* value
			Low stable group *n* = 800	Medium growth group *n* = 749	High growth group *n* = 629		
Age, years	Median (P_25_, P_75_)	57 (51,63)	57 (52,63)	58 (51,63)	57 (51,63)	3.010^a^	0.222
	Mean ± SE	57.60 ± 0.17	57.65 ± 0.28	57.89 ± 0.28	57.19 ± 0.31		
Gender, *n* (%)	Male	1105 (50.73)	504 (45.61)	374 (33.85)	227 (20.54)	102.322^b^	<0.001
	Female	1073 (49.27)	296 (27.59)	375 (34.94)	402 (37.47)		
Residence, *n* (%)	Rural	1778 (81.63)	588 (33.07)	623 (35.04)	567 (31.89)	66.871^b^	<0.001
	Urban	400 (18.37)	212 (53.00)	126 (31.50)	62 (15.50)		
Education, *n* (%)	Primary school and below	1368 (62.81)	397 (29.02)	498 (36.40)	473 (34.58)	110.839^b^	<0.001
	Junior high school	541 (24.84)	254 (46.95)	174 (32.16)	113 (20.89)		
	High school and above	269 (12.35)	149 (55.39)	77 (28.62)	43 (15.99)		
Marital status, *n* (%)	Single	403 (18.50)	105 (26.06)	143 (35.48)	155 (38.46)	31.238^b^	<0.001
	Married	1775 (81.50)	695 (39.15)	606 (34.14)	474 (26.71)		
Health insurance status, *n* (%)	No	90 (4.13)	33 (36.67)	36 (40.00)	21 (23.33)	1.859^b^	0.395
	Yes	2088 (95.87)	767 (36.73)	713 (34.15)	608 (29.12)		
Social activity participation, *n* (%)	No	970 (44.54)	311 (32.06)	349 (35.98)	310 (31.96)	17.407^b^	<0.001
	Yes	1208 (55.46)	489 (40.48)	400 (33.11)	319 (26.41)		
Number of chronic diseases, *n* (%)	1	996 (45.73)	430 (43.17)	343 (34.44)	223 (22.39)	65.222^b^	<0.001
	2	630 (28.93)	222 (35.24)	223 (35.40)	185 (29.36)		
	≥3	552 (25.34)	148 (26.81)	183 (33.15)	221 (40.04)		
Physical activity, *n* (%)	No	1272 (58.40)	458 (36.01)	446 (35.06)	368 (28.93)	0.843^b^	0.656
	Yes	906 (41.60)	342 (37.75)	303 (33.44)	261 (28.81)		
Smoking status, *n* (%)	Never	1295 (59.46)	432 (33.36)	446 (34.44)	417 (32.20)	23.675^b^	<0.001
	Former	211 (9.69)	94 (44.55)	73 (34.60)	44 (20.85)		
	Current	672 (30.85)	274 (40.77)	230 (34.23)	168 (25.00)		
Drinking status, *n* (%)	No	1993 (91.51)	725 (36.38)	683 (34.27)	585 (29.35)	2.714^b^	0.257
	yes	185 (8.49)	75 (40.54)	66 (35.68)	44 (23.78)		
BMI, kg/m^2^	Median (P_25,_ P_75_)	24 (21,27)	24. (22,27)	24 (21,26)	23 (21,26)	33.821^a^	<0.001
	Mean ± SE	24.13 ± 0.09	24.71 ± 0.14	23.90 ± 0.14	23.67 ± 0.17		
Midday napping, minutes	Median (P_25_, P_75_)	30 (0,60)	40 (0,60)	30 (0,60)	0 (0,60)	24.910^a^	<0.001
	Mean ± SE	38.38 ± 0.94	44.26 ± 1.62	36.75 ± 1.52	33.14 ± 1.71		
Nighttime sleep duration, hours	Median (P_25_, P_75_)	6 (5,8)	7 (6,8)	6 (5,7)	6 (4,7)	122.688^a^	<0.001
	Mean ± SE	6.31 ± 0.04	6.80 ± 0.05	6.27 ± 0.06	5.73 ± 0.08		

^aThe Kruskal-Wallis H test.^

^bThe Pearson χ2 test.^

### 3.4 Selection of the multinomial logistic regression model for analyzing depression trajectories

Multinomial logistic regression model 1: number of chronic diseases, 1 predictor as independent variable, trajectory subgroups as dependent variable. The reason for building the model was to investigate the relationship between chronic disease and depressive trajectories. Multinomial logistic regression model 2: gender, residence, education, marital status, social activity participation, number of chronic diseases, smoking, BMI, midday napping, nighttime sleep duration, the above mentioned 10 predictors as independent variable, trajectory subgroups as dependent variable. The reason behind the construction of this model was that the aforementioned independent variables exert an influence on each trajectory. Multinomial logistic regression model 3: age, gender, residence, education, marital status, social activity participation, number of chronic diseases, smoking status, BMI, midday napping, nighttime sleep duration, with the above 11 predictors as independent variables and trajectory subgroups as the dependent variable. Although age is not a determining factor for each trajectory, it is nevertheless important to observe the model’s performance after incorporating this variable. Multinomial logistic regression model 4: age, gender, drinking status, health insurance status, physical activity, residence, education, marital status, social activity participation, number of chronic diseases, smoking status, BMI, midday napping, nighttime sleep duration, the above 14 predictors as independent variables and trajectory subgroups as dependent variable were compared.

Since the smaller the value of the AIC, −2loglikelihood, indicates that the selected model fits the observed data better, and the fewer the number of parameters used in the model, the better its performance in solving the overfitting problem, model 3 was finally selected (Table 3).

**Table 3:** The selection of optimal multinomial logistic regression model for the subgroups of depression trajectories.

Model	Model fitting criteria			Likelihood ratio tests		
	AIC	-2LogLikelihood	Number of parameters	*χ* ^2^	df	*P* value
Model1	4707.48	40.31	1	64.82	4	<0.001
Model2	4337.49	4302.09	10	461.87	26	<0.001
Model3	4332.36	4293.38	11	470.57	28	<0.001
Model4	4341.70	4290.83	14	473.12	34	<0.001

Multinomial logistic regression model 1: number of chronic diseases, 1 predictor as independent variable, trajectory subgroups as dependent variable. Multinomial logistic regression model 2: gender, residence, education, marital status, social activity participation, number of chronic diseases, smoking, BMI, midday napping, nighttime sleep duration, the above mentioned 10 predictors as independent variable, trajectory subgroups as dependent variable. Multinomial logistic regression model 3: age, gender, residence, education, marital status, social activity participation, number of chronic diseases, smoking status, BMI, midday napping, nighttime sleep duration, with the above 11 predictors as independent variables and trajectory subgroups as the dependent variable. Multinomial logistic regression model 4: age, gender, drinking status, health insurance status, physical activity, residence, education, marital status, social activity participation, number of chronic diseases, smoking status, BMI, midday napping, nighttime sleep duration, the above 14 predictors as independent variables and trajectory subgroups as dependent variable.

### 3.5 Logistic regression analysis of factors influencing depression trajectories

**Figure 4:**
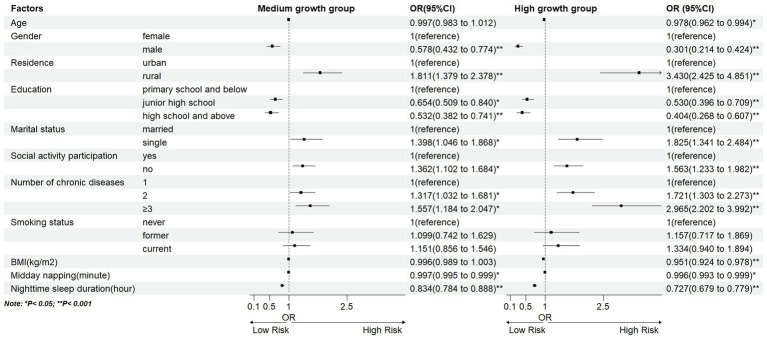
Longitudinal LCMM based depression trajectories using CESD-10 score. Multinomial logistic regression model 3: age, gender, residence, education, marital status, social activity participation, number of chronic diseases, smoking status, BMI, midday napping, nighttime sleep duration, with the above 11 predictors as independent variables and trajectory subgroups as the dependent variable.

Model 3 was used to perform multinomial logistic regression analyses. Using the ‘Low stable group’ as the reference group (Supplementary Table S2). In the target population of our study, which comprised the middle-aged and older Chinese individuals with chronic diseases, the results indicated that: female, rural, primary school and below, single, no social activity participation, suffering from multiple chronic diseases, and the shorter midday napping and nighttime sleep duration were more likely to be in the “Medium growth group”; and the younger the age, female, rural, primary school and below, single, social activity participation (no), suffering from multiple chronic diseases, shorter midday napping and nighttime sleep duration, and lower BMI were to be in the “High growth group” (Figure 4).

### 3.6 Sensitivity analysis

**Figure 5:**
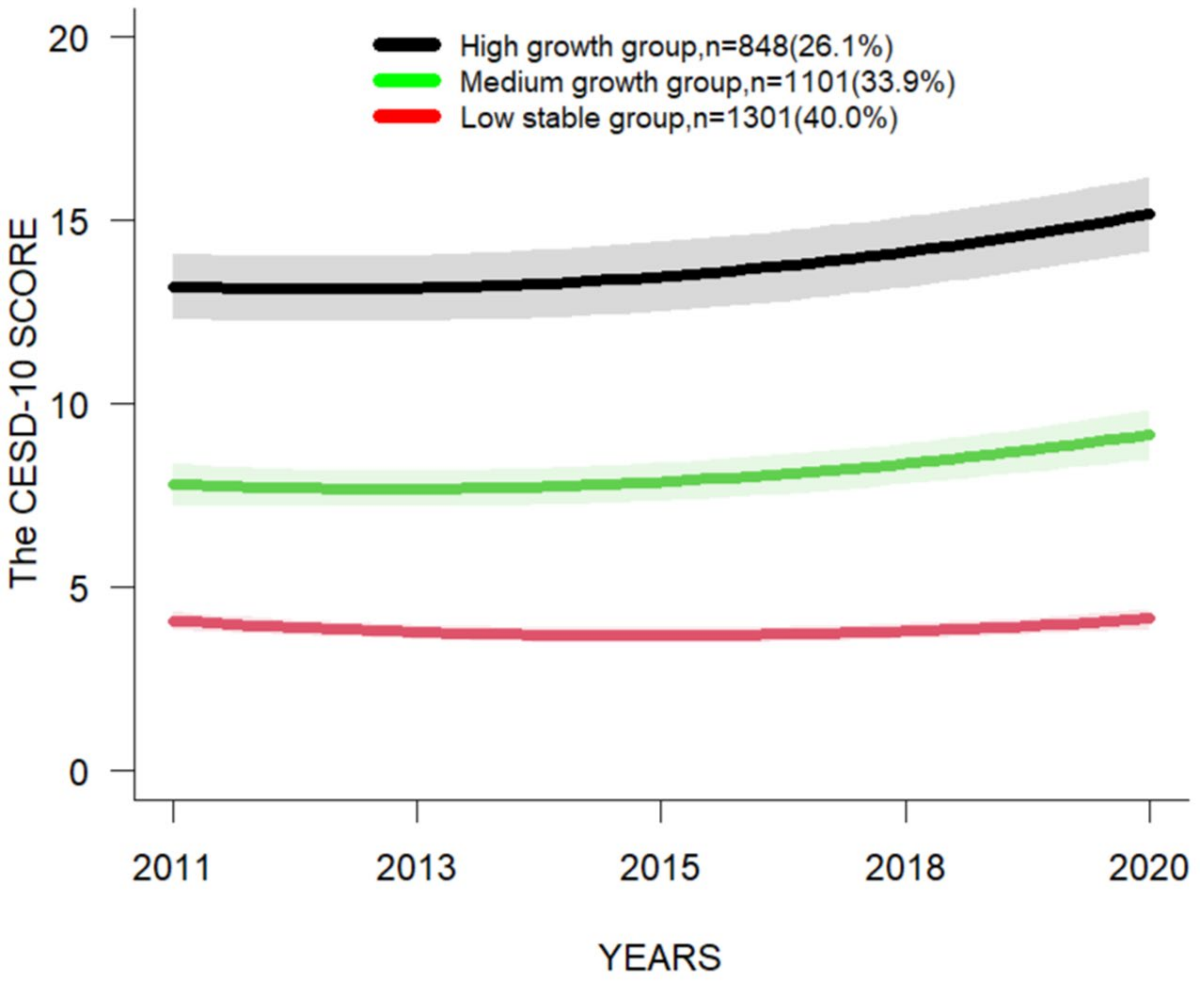
Longitudinal LCMM based depression trajectories using CESD-10 score (sensitive analysis).

The trajectory of depression symptoms calculated through the sensitivity analysis of participants undergoing five rounds of depression measurements was analogous to the trajectory in the main analysis (Figure 5). Utilizing this trajectory classification, we further executed the multinomial logistic regression and discovered that the estimates were comparable to those in the main analysis (Supplementary Table S3).

## 4 Discussion

### 4.1 Three subgroups of depression trajectories were identified

Three subgroups of depression trajectories were identified, called “Low stable group,” “Medium growth group” and “High growth group.” In this study, 34.4% of the middle-aged and older individuals with chronic diseases are categorized into the “Low stable group.” The mean of CESD-10 score is consistently low across all time points, and the prevalence of depression does not exceed 10%, indicating relatively low levels of depression; 36.7% of the middle-aged and older individuals with chronic diseases are categorized into the “Medium growth group.” The mean of CESD-10 score shows an overall upward trend at various time points. However, the mean of score does not exceed 10. The prevalence of depression is under 50%, indicating the presence of moderately levels of depression; 28.9% of the middle-aged and older individuals with chronic diseases are categorized into the “High growth group.” Both the mean of score and the prevalence of depression at various time points have demonstrated higher levels, with the mean of score exceeding 10 and the prevalence all surpassing 70%, which suggests that this group may exhibit more severe clinical symptoms of depression. A study by Xiang (30) has indicated that the initial severity of depressive symptoms, as well as their persistence, are predictors of both mild and severe depression. Accordingly, it is recommended that the “Medium growth group” and the “High growth group” be given priority when developing strategies for the prevention and treatment of depression. Early screening and targeted interventions are recommended to improve depressive rates, with a particular focus on understanding the factors influencing the three aforementioned trajectories. These findings will facilitate the establishment of depression screening and intervention programs for the middle-aged and older individuals in China.

### 4.2 The factors between different trajectory subgroups were analyzed

We found that the middle-aged and older individuals with chronic disease who were female, rural, primary school and below, single, had no social activity participation, suffering from multiple chronic diseases, and the shorter midday napping and nighttime sleep duration, were more likely to have higher and increasing levels of depression. Previous studies have demonstrated that the prevalence of depression in older Chinese females is higher than that in males (31). This may be related to the physical characteristics of females themselves. Physiologically, the middle-aged and older females are prone to irritability, anxiety and other emotions due to the physiological changes associated with menopause, which may lead to depressive symptoms (14). This is explored in greater detail by Slavich et al. (32) in their commentary, where fluctuations in women’s ovarian hormone levels exert an influence on women’s brain structure and function, susceptibility to stress, and the body’s inflammatory response, which have been associated with risk factors for depression. Our study found that depression severity might be lower in urban areas than in rural areas, which is consistent with previous research (33). Those residing in rural areas are subject to a number of socioeconomic pressures and are more likely to experience depression due to the limited availability of health resources. However, a recent study by Wang et al. (34) based on CHARLS data found that urbanization may have no effect on depression in older adults. The influence of regional factors on depression still needs further study. People with lower education levels may face greater problems with depression (35). It can be illustrated that Socioeconomic status frequently influences an individual’s access to educational resources (36). Studies have indicated that a low socioeconomic status is frequently correlated with a heightened risk of developing depression (37). This connection partially accounts for why individuals with lower educational levels might be more susceptible to depression. Nevertheless, it is crucial to note that this relationship is intricate, and the mechanisms through which educational level and depressive symptoms arise need to be clarified (38). Among the middle-aged and older individuals with chronic diseases, lack of a spouse or social behavior may lead to increased levels of depression, which is often due to a lack of social support and the small social networks (39). Multiple studies have shown a positive correlation between the number of chronic diseases and the severity of depression (12, 14). Our results also support this conclusion. A study by Jiang et al. (40) examined the prevalence of depression in middle-aged and older Chinese individuals with chronic diseases. They found that chronic diseases (such as diabetes, stroke and arthritis) were closely related to the occurrence of depression in the middle-aged and older Chinese individuals with chronic diseases. Furthermore, the study found that chronic diseases were directly proportional to depression risk, with a higher quantity of chronic diseases suggesting higher depression rates (40). A meta-analysis by Read et al. (41) found that the risk of depression among the multiple-patient groups was 2.97 times that of the healthy group. In addition, a study by Guo et al. (42) also suggests that people with multiple chronic conditions are more likely to suffer from depression, which supports our findings. We must emphasize the combined impact of multiple diseases on the overall health and quality of life of patients. Midday napping is a common practice among the Chinese population, particularly among the older adults, and has been regarded as a healthy lifestyle choice (43). A study of Chinese middle-aged and older individuals with depressive disorders by Li et al. (44) revealed that those who slept for longer periods exhibited a greater likelihood of symptom remission. Furthermore, a study by Dong et al. (45) conducted in the adult population in the United States has demonstrated that both insufficient and excessive sleep duration is associated with an increased risk of depression in adults. In contrast, the research by Zhong et al. (46) among the older Chinese adults has demonstrated that individuals who slept for more hours at night exhibited lower levels of depressive symptoms than those who slept for fewer hours. The findings of that study are similarly reflected in this current study, as our “Low stable group” (associated with low levels of depression) comprised of individuals who slept for longer hours. This emphasizes the necessity for further research into the potential benefits of extending sleep duration in older adults, with the aim of preventing the onset of depression. In addition to the above factors, we also discovered that among the middle-aged and older Chinese individuals with chronic diseases, the risk of depression decreases with increasing age. The phenomenon may be more closely associated with the significance of quality of life and mental health issues in the middle-aged population of individuals with chronic conditions (47). In contrast, the individuals in their middle age are confronted with the dual challenges of balancing career and family responsibilities, as well as navigating through significant life transitions such as children becoming independent and aging parents, all of which may collectively elevate the susceptibility to depression (48). The lower the BMI of the population was more likely to be in the high level of depression increase group. A study by Fang et al. (49) has indicated that obesity may have a significant inhibitory effect on depression, which is consistent with our findings. Besides, a study by O’Loughlin et al. (50) has indicated that higher BMI is associated with a lower risk of depression in East Asian populations living in China, which may be related to the traditional Chinese belief that obesity is a positive because only the rich can afford to put on weight by eating food.

### 4.3 The health insurance status and drinking status were not factors in each trajectory

In our analysis of baseline characteristics across trajectory subgroups, we found that health insurance status, drinking status, and physical activity were not factors influencing the trajectory of depression among the middle-aged and older individuals with chronic diseases. One possible reason for this is that having health insurance can indeed provide a channel for accessing medical resources and economic security, thereby alleviating the economic pressure caused by medical expenses. However, the chronic illness is a long-term, persistent condition that accompanies individuals at various stages throughout their lives. The insured individuals with chronic diseases may encounter numerous challenges, such as persistent pain, functional limitations, and reduced quality of life, all of which can significantly contribute to the development of depression in individuals. A study by Lu (51) revealed a significant negative correlation between survey respondents’ level of satisfaction with the Medicare program and the occurrence of depression. Therefore, we should not only focus on whether the middle-aged and older individuals with chronic disease in China have medical insurance, but also pay more attention to their satisfaction with medical insurance. Our study shows that alcohol consumption is not a significant influence across the three subgroups of the depression trajectory. This finding provides new evidence to explore the complex relationship between alcohol use and depression. A study by Schouten et al. (52) indicates that in the general population, alcohol consumption does not predict the persistence of major depressive disorder (MDD) after 3 years. However, a study among the middle-aged and older Chinese by Zhu et al. (53) found a causal relationship between alcohol consumption and a reduced risk of depression. Another study by Li et al. (54) has shown that alcohol use disorders are associated with an increased risk of subsequent depressive symptoms. The differences between these findings highlight the complexity of the relationship between alcohol use and depression, while also highlighting the need for more nuanced and in-depth interpretation.

### 4.4 The role of physical activity in ameliorating depression needs to be further explored

As research into treatments for depression continues, it appears that physical activity may be an effective method of aiding in the treatment of depression. Many scholars have explored in depth the mechanisms by which physical activity affects depressive symptoms from the physiological and psychological (55). In terms of physiological factors, Depression is associated with brain structure and function, inflammation and oxidative stress in the body and hormone secretion, and regular exercise helps to maintain normal brain structure and function, resist inflammation and oxidative stress response, regulate hormone secretion, and thus reduce the risk of depression (56); In terms of psychological factors, physical self-worth and elf-perceived physical condition can be improved through exercise, which in turn can alleviate depressive symptoms (57). Besides, there is a relationship between self-efficacy and depression. A study by Haller et al. (58) showed that physical exercise improved depression symptoms along with increased self-efficacy, and a study by suggested that self-efficacy may be a mediating factor between physical exercise and depression (59). Additionally, a population-based, randomized-controlled study of 20 adolescent inpatients showed a reduction in depression symptoms when structured physical exercise therapy was included in their care plan (60). In the population of adults, it is believed that physical activity may serve to reduce the risk of developing depression (61). The aged with depression often have multiple co-morbidities and cognitive impairments, which makes their situation even more complicated (62). A study of the middle-aged and older individuals with Parkinson’s disease by Wu et al. (63), physical activity has been shown to alleviate depressive symptoms. While this finding provides valuable insight into the role of physical exercise in alleviating depression in individuals with specific chronic conditions, it must be noted that this study was limited to individuals with Parkinson’s disease. Therefore, the results may not apply to the broader population of the middle-aged and older individuals with chronic diseases. In our study, the analysis of the three subgroups of depression trajectories found that physical exercise was not a significant factor affecting each trajectory. It is concluded that the reason may be that chronic diseases may affect the individual mobility of older people (64). Although our findings do not support the utility of physical activity in improving depressive symptoms in middle-aged and older individuals with chronic diseases, possibly due to the methodological and sample selection limitations. However, we should not lose sight of the value of physical activity as an important component of healthy aging. Physical activity plays a key role in preventing or reducing falls, pain, sarcopenia, osteoporosis and cognitive impairment (65). Hence, we still strongly recommend that appropriate physical activity programs should be developed for the individuals with chronic diseases.

### 4.5 Strengths and limitations

This research has several advantages. Firstly, it extends the understanding of the relationship between the middle-aged and older Chinese individuals with chronic diseases and their subgroups of depression trajectories in the context of Chinese socio-economic and cultural contexts. Secondly, based on LCMM, the three subgroups of depression trajectories were identified in this research. The characteristics and trends of different trajectory subgroups assist in the development of detailed intervention and support strategies to improve depressive mood in the middle-aged and older individuals with chronic diseases.

This research has several weaknesses. Firstly, this study is based on a large population study of middle-aged and older adults in China, and it is important to recognize that cultural, social, and health care system differences may limit the direct generalization of these results to other populations. Future cross-cultural studies will help explore the consistency of these findings across different populations and identify potentially universal and culture-specific factors that influence depressive symptoms in older adults. Secondly, it focuses on the impact of the number of chronic diseases on depression. It does not delve into the influence of specific chronic disease types, the interplay between different types of chronic diseases, and their collective impact on depression. This is an area that warrants further investigation. Thirdly, the social activity participation has not arrived at a consensus on the notion of social activity participation. We merely judge whether the respondents participated in social activities in the past month based on the questions of the CHARLS questionnaire (66). If the subsequent research can clearly define the social activity participation of middle-aged and older adults in China, it will be more beneficial to explore the relationship between depression and social activities within this population. Fourthly, there is a complex correlation between depression and various factors in Chinese middle-aged and older patients with chronic diseases. For example, studies have pointed out that depression is also closely related to psychological characteristics and physical health (67, 68), but we have not discussed it in depth. Therefore, future studies should include more factors in Chinese middle-aged and older individuals with chronic diseases to explore their correlation with depression, or adopt different analytical strategies to further explore the correlation between a certain factor and depression. Finally, the information collected through questionnaires may have reporting bias, so other objective indicators should be used to measure the incidence of depression and its risk factors in the middle-aged and older individuals with chronic diseases.

## 5 Conclusion

In conclusion, this study provides new insights into the heterogeneous trajectory of depression in middle-aged and older Chinese patients with chronic diseases. Our analysis revealed three distinct depression tracks: the “Low stable group,” the “Medium growth group,” and the “High growth group.” These findings highlight the complexity and dynamics of depressive symptoms in this population. These tracks have the potential to help predict different risk factors and more accurately differentiate high-risk populations in order to provide more effective monitoring and intervention. In addition, we also highlight areas that require further research, such as physical activity and the relationship between alcohol use and depression in middle-aged and older adults with chronic diseases.

## Data Availability

The datasets presented in this study can be found in online repositories. The names of the repository/repositories and accession number (s) can be found at: http://charls.pku.edu.cn.
