# Supplementary material for: Identifying the subgroups of depression trajectories among the middle-aged and older Chinese individuals with chronic diseases: an 8-year follow-up study based on CHARLS
Source: Front Public Health. 2024 Sep 11;12:1428384. doi: 10.3389/fpubh.2024.1428384 (PMC11524047; doi:10.3389/fpubh.2024.1428384)
Supplement: Supplementary file 1 [file Data_Sheet_1.docx]

**Appendix 1 The code for all variables and their problem descriptions**

| **Variable** | **Code** | **Question description** | **Answer** |
| --- | --- | --- | --- |
| Gender | BA000_W2_3 | Interviewer record the Respondent’s gender | 1. Male  2. Female |
| Age | BA002 | What’s your actual date of birth? |  |
| Residence | A001 | Take down the type of this neighborhoods. | 1.Rural Village  2.Urban Community |
| Education | BD001_W2_4 | What’s the highest level of education your have now (not including adult education)? | 1. No formal education (illiterate)  2. Did not finish primary school  3. Sishu/home school  4. Elementary school  5. Middle school  6. High school  7. Vocational school  8. Two-/Three-Year College/Associate degree  9. Four-Year College/Bachelor’s degree  10. Master’s degree  11. Doctoral degree/Ph.D. |
| Marital status | BE001 | What is your marital status? | 1. Married and live with spouse  2. Married but don’t living with spouse temporarily for reasons such as work  3. Separated, don’t live together as a couple anymore  4. Divorced  5. Widowed  6. Never married |
| Health insurance status | EA001_W4 | Are you the policy holder/primary beneficiary of any of the types of health insurance listed below? (circle all that apply) | 1. Urban employee medical insurance (yi-bao)  2. Urban and rural resident medical insurance (integrated urban resident medical insurance and new rural cooperative medical insurance)  3. Urban resident medical insurance  4. New rural cooperative medical insurance (he-zuo-yi-liao)  5. Government medical insurance  6. Medical aid  7. Private medical insurance: purchased by work unit  8. Private medical insurance: purchased by individual  9. Urban non-employed persons’s health insurance  10. Long-term care insurance  11. Other medical insurance  12. No insurance |
| Physical activity | DA051 | Now we would like to ask about the amount of time you spend on different types of physical activities in a usual week. | 1.Now, think about all the vigorous activities requiring hard/high-intensity physical effort that you do in a usual week. Vigorous activities make you breathe much harder than normal and may include heavy lifting, digging, plowing, aerobics, fast bicycling, and cycling with a heavy load. Think only about those physical activities that you did for at least 10 minutes at a time.  2.Now think about activities which take moderate physical effort that you do in a usual week. Moderate physical activities make you breathe somewhat harder than normal and may include carrying light loads, bicycling at a regular pace, or mopping the floor. Again, think about only those physical activities that you did for at least 10 minutes at a time.  3.Now think about the time you spend walking in a usual week. This includes at work and at home, walking to travel from place to place, and any other walking that you might do solely for recreation, sport, exercise, or leisure. |
| Social activity participation | DA056 | Have you done any of these activities in the last month? (Check all that apply) | 1. Interacted with friends  2. Played Ma-jong, played chess, played cards, or went to community club  3. Provided help to family, friends, or neighbors who do not live with you  4. Went to a sport, social, or other kind of club  5. Took part in a community-related organization  6. Done voluntary or charity work  7. Cared for a sick or disabled adult who does not live with you  8. Attended an educational or training course  9. Stock investment  10. Used the Internet  11. Other  12. None of these |
| Smoking status | DA059 | Have you ever chewed tobacco, smoked a pipe, smoked self-rolled cigarettes, or smoked cigarettes/cigars? | 1. Yes  2. No |
|  | DA061 | Do you still have the habit or have you totally quit? | 1. Still have  2. Quit |
| Drinking status | DA067 | Did you drink any alcoholic beverages, such as beer, wine, or liquor in the past year? How often? | 1. Drink more than once a month  2. Drink but less than once a month  3. None of these |
| Nighttime sleep duration (hours) | DA049 | During the past month, how many hours of actual sleep did you get at night (average hours for one night)? |  |
| Midday napping (minutes) | DA050 | During the past month, how long did you take a nap after lunch? |  |
| Height | PI001 | Can you understand the measurement method and are you willing to participate in this measurement? | 1. YES  2. No |
| Weight | PL002 | Can you understand the measurement method and are you willing to participate in this measurement? | 1. Yes  2. No |
| Chronic disease | DA007 | Have you been diagnosed with [conditions listed below, read one by one] by a doctor? | 1. Hypertension  2. Dyslipidemia  3. Diabetes or high blood sugar  4. Cancer or malignant tumor  5. Chronic lung diseases  6. Liver disease  7. Heart attack  8. Stroke  9. Kidney disease  10. Stomach or other digestive diseases  11. Memory-related disease  12. Arthritis or rheumatism  13. Asthma |
| Depression | DC009 | I was bothered by things that don’t usually bother me | 1. Rarely or none of the time  2. Some or a little of the time  3. Occasionally or a moderate amount of the time  4. Most or all of the time  8. Do not know  9. Refuse to answer |
|  | DC010 | I had trouble keeping my mind on what I was doing | 1. Rarely or none of the time  2. Some or a little of the time  3. Occasionally or a moderate amount of the time  4. Most or all of the time  8. Do not know  9. Refuse to answer |
|  | DC011 | I felt depressed | 1. Rarely or none of the time  2. Some or a little of the time  3. Occasionally or a moderate amount of the time  4. Most or all of the time  8. Do not know  9. Refuse to answer |
|  | DC012 | I felt everything I did was an effort | 1. Rarely or none of the time  2. Some or a little of the time  3. Occasionally or a moderate amount of the time  4. Most or all of the time  8. Do not know  9. Refuse to answer |
|  | DC013 | I felt hopeful about the future | 1. Rarely or none of the time  2. Some or a little of the time  3. Occasionally or a moderate amount of the time  4. Most or all of the time  8. Do not know  9. Refuse to answer |
|  | DC014 | I felt fearful |  |
|  | DC015 | My sleep was restless |  |
|  | DC016 | I was happy |  |
|  | DC017 | I felt lonely |  |
|  | DC018 | I could not get “going” |  |
|  |  |  |  |
|  |  |  |  |
|  |  |  |  |
|  |  |  |  |
