# Supplementary material for: Identifying the subgroups of depression trajectories among the middle-aged and older Chinese individuals with chronic diseases: an 8-year follow-up study based on CHARLS
Source: Front Public Health. 2024 Sep 11;12:1428384. doi: 10.3389/fpubh.2024.1428384 (PMC11524047; doi:10.3389/fpubh.2024.1428384)
Supplement: Supplementary file 2 [file Data_Sheet_2.docx]

Supplementary Material

Identifying the subgroups of depression trajectories among the middle-aged and elderly Chinese individuals with chronic diseases: an 8-year follow-up study based on CHARLS

Jiaxing Pei^1,2^, Mei Hu^3^, Qiang Lu^1^, Pengfei Zhou^4^, Yijing Shang^1,2^, Huiwang Zhang^2^, Xiaoguang Yang^1*^, Yunming Li^1,2*^

*** Correspondence:**Corresponding Author
Xiaoguang Yang: [52172999@qq.com](mailto:52172999@qq.com); Yunming Li: [lee3082@sina.com](mailto:lee3082@sina.com)

# Supplementary Figure

**Baseline (2011) include participants=17,705**

**Complete follow-up from 2011 to 2020, n=5,395**

**Sample, n=3,303**

**Excluded samples:**

**Populations lacking sociodemographic information also lack information on depression surveys, n=2,092**

**Final Sample, n=2,178**

**Excluded samples:**

**No chronic diseases, n=1,103**

**Age <45, n=22**

**Figure 1.** Process of sample population screening


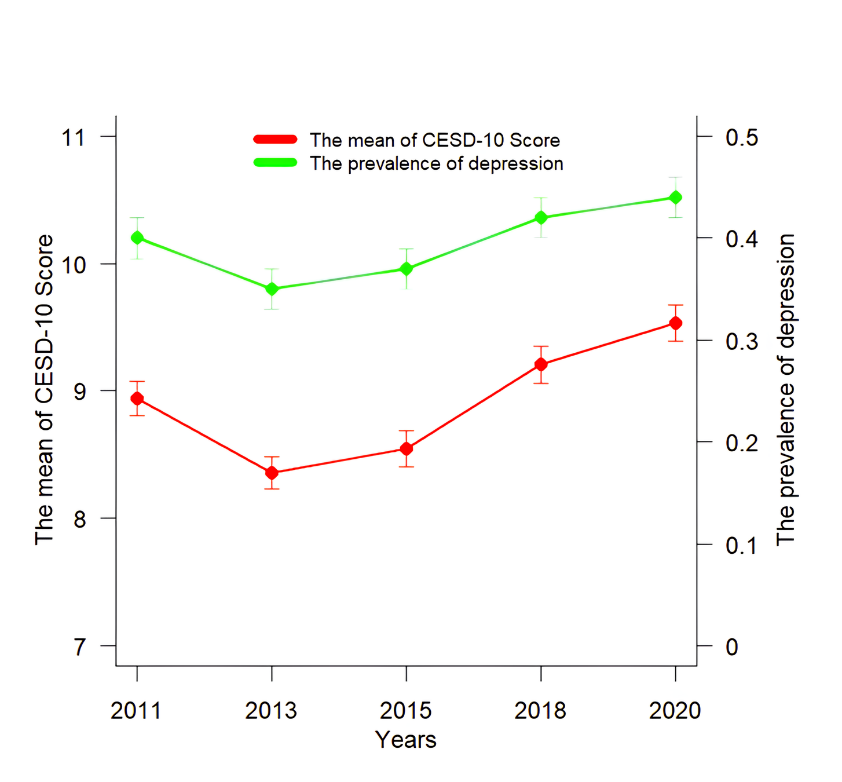


**Figure 2.** The mean of CES-D score (mean ± standard error) across 5 data collections during 2011-2020; prevalence (*p* 95% CI)


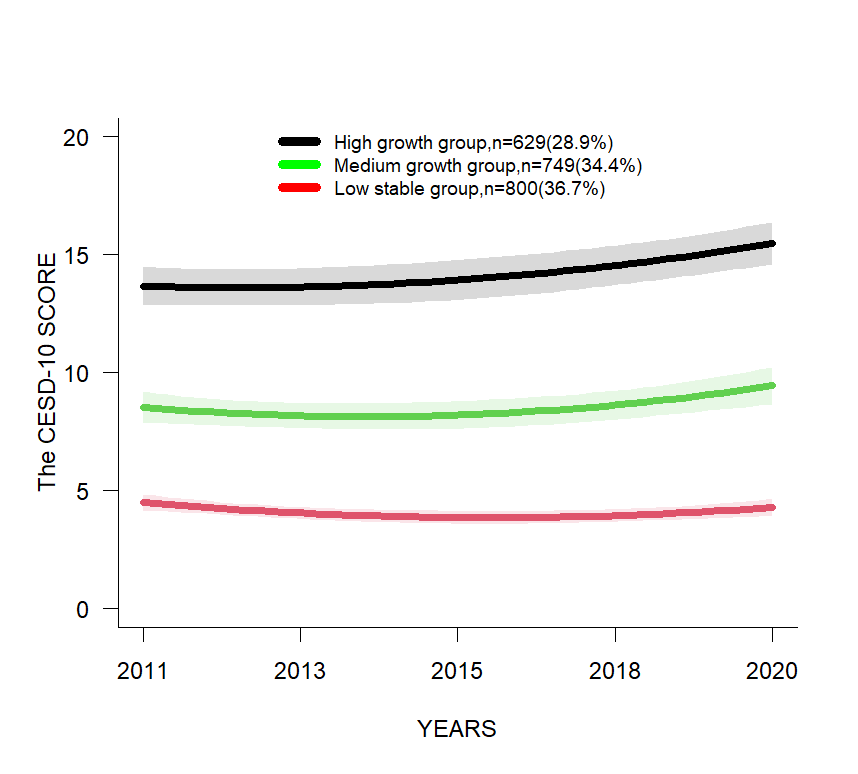


**Figure 3.** Longitudinal LCMM based depression trajectories using CESD-10 score


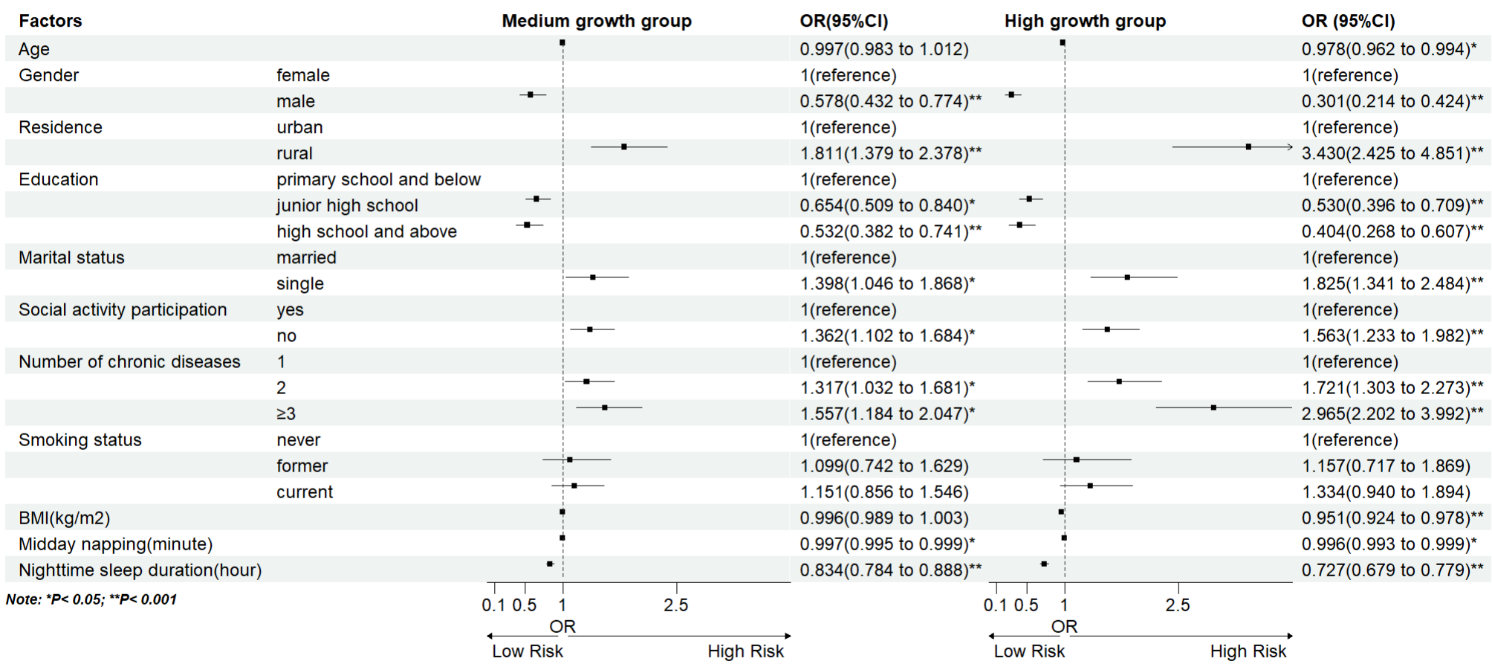


**Figure 4.** Longitudinal LCMM based depression trajectories using CESD-10 score

Note: Multinomial logistic regression model 3: age, gender, residence, education, marital status, social activity participation, number of chronic diseases, smoking status, BMI, midday napping, nighttime sleep duration, with the above 11 predictors as independent variables and trajectory subgroups as the dependent variable.


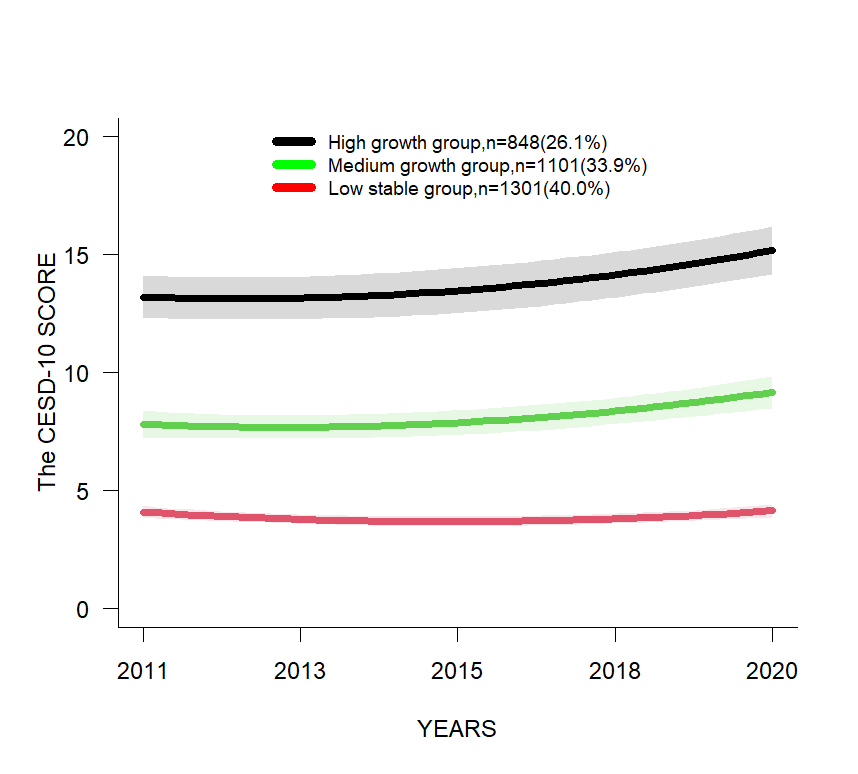


**Figure 5.** Longitudinal LCMM based depression trajectories using CESD-10 score (Sensitive Analysis).
