# Supplementary material for: Identifying the subgroups of depression trajectories among the middle-aged and older Chinese individuals with chronic diseases: an 8-year follow-up study based on CHARLS
Source: Front Public Health. 2024 Sep 11;12:1428384. doi: 10.3389/fpubh.2024.1428384 (PMC11524047; doi:10.3389/fpubh.2024.1428384)
Supplement: Supplementary file 3 [file Table_1.DOCX]

**Table S1. Goodness of fit indices of models with latent classes**

| Trajectories for CESD-10 Depression | CONV | AIC | BIC | SABIC | Entropy | Proportion of each class (%) | | | | Mean of posterior probabilities in each class (%) | | | | Posterior probabilities >0.7 (%) | | | |
| --- | --- | --- | --- | --- | --- | --- | --- | --- | --- | --- | --- | --- | --- | --- | --- | --- | --- |
|  |  |  |  |  |  | 1 | 2 | 3 | 4 |  |  |  |  |  |  |  |  |
| 1 | 1 | 67330.1 | 67387.0 | 67355.2 | 1.0 | 100.0 |  |  |  |  | | | |  | | | |
| 2 | 1 | 66759.5 | 66844.8 | 66797.2 | 0.6 | 51.4 | 48.6 |  |  | 92.0 | 84.8 |  |  | 88.9 | 85.8 |  |  |
| 3 | 1 | 66628.6 | 66742.3 | 66678.7 | 0.6 | 28.9 | 36.7 | 34.4 |  | 88.1 | 84.3 | 69.3 |  | 83.6 | 81.1 | 53.1 |  |
| 4 | 1 | 66615.6 | 66757.8 | 66678.3 | 0.6 | 9.4 | 23.5 | 34.8 | 32.4 | 81.1 | 61.7 | 83.5 | 77.4 | 70.7 | 19.6 | 79.0 | 68.9 |

**Note:** convergence criterion (1 = converged); AIC (the lower the better); BIC (the lower the better); SABIC (the lower the better); Entropy (the closer to one the better).
